# Mitochondria-Targeted Catalase Reverts the Neurotoxicity of hSOD1^G93A^ Astrocytes without Extending the Survival of ALS-Linked Mutant hSOD1 Mice

**DOI:** 10.1371/journal.pone.0103438

**Published:** 2014-07-23

**Authors:** Mariana Pehar, Gyda Beeson, Craig C. Beeson, Jeffrey A. Johnson, Marcelo R. Vargas

**Affiliations:** 1 Department of Cell and Molecular Pharmacology and Experimental Therapeutics, Medical University of South Carolina, Charleston, South Carolina, United States of America; 2 SCCP Drug Discovery and Biomedical Sciences, Medical University of South Carolina, Charleston, South Carolina, United States of America; 3 Division of Pharmaceutical Sciences, Waisman Center, Molecular and Environmental Toxicology Center, University of Wisconsin, Madison, Wisconsin, United States of America; Inserm, France

## Abstract

Dominant mutations in the Cu/Zn-superoxide dismutase (SOD1) cause familial forms of amyotrophic lateral sclerosis (ALS), a fatal disorder characterized by the progressive loss of motor neurons. The molecular mechanism underlying the toxic gain-of-function of mutant hSOD1s remains uncertain. Several lines of evidence suggest that toxicity to motor neurons requires damage to non-neuronal cells. In line with this observation, primary astrocytes isolated from mutant hSOD1 over-expressing rodents induce motor neuron death in co-culture. Mitochondrial alterations have been documented in both neuronal and glial cells from ALS patients as well as in ALS-animal models. In addition, mitochondrial dysfunction and increased oxidative stress have been linked to the toxicity of mutant hSOD1 in astrocytes and neurons. In mutant SOD1-linked ALS, mitochondrial alterations may be partially due to the increased association of mutant SOD1 with the outer membrane and intermembrane space of the mitochondria, where it can affect several critical aspects of mitochondrial function. We have previously shown that decreasing glutathione levels, which is crucial for peroxide detoxification in the mitochondria, significantly accelerates motor neuron death in hSOD1^G93A^ mice. Here we employed a catalase targeted to the mitochondria to investigate the effect of increased mitochondrial peroxide detoxification capacity in models of mutant hSOD1-mediated motor neuron death. The over-expression of mitochondria-targeted catalase improved mitochondrial antioxidant defenses and mitochondrial function in hSOD1^G93A^ astrocyte cultures. It also reverted the toxicity of hSOD1^G93A^-expressing astrocytes towards co-cultured motor neurons, however ALS-animals did not develop the disease later or survive longer. Hence, while increased oxidative stress and mitochondrial dysfunction have been extensively documented in ALS, these results suggest that preventing peroxide-mediated mitochondrial damage alone is not sufficient to delay the disease.

## Introduction

Amyotrophic lateral sclerosis (ALS) is caused by the progressive degeneration of motor neurons in the spinal cord, brain stem, and motor cortex. Motor neuron death leads to muscle weakness and paralysis causing death in one to five years from the time of symptoms onset. In the United States and the United Kingdom, ALS accounts for about 1 in 500 to 1 in 1,000 adult deaths [1]. Most ALS cases are sporadic (SALS) and exposure to yet unidentified environmental toxicants might be responsible for SALS. About 5–10% of ALS cases are inherited (familial ALS, FALS) and the first ALS-linked gene identified was superoxide dismutase 1 (SOD1) [2]. SOD1 mutations account for up to 20% of FALS and 1–2% of apparently SALS. Several other genes have now been identified in many FALS pedigrees [3], [4]. Each mutated gene has its own genetic and molecular signature, but FALS and SALS are phenotypically indistinguishable. However, a significant share of our understanding of the disease come from the study of rodent models over-expressing ALS-linked mutant SOD1, which develop an ALS-like phenotype [5]. The molecular mechanism underlying the toxic gain-of-function of mutant hSOD1s remains uncertain, however several lines of evidence suggest that toxicity to motor neurons requires damage to non-neuronal cells [6]–[8]. In line with this observation, primary astrocytes isolated from mutant hSOD1 over-expressing rats or mice induce motor neuron death in co-culture [9], [10].

Mitochondrial alterations have been documented in both neuronal and glial cells from ALS patients as well as in ALS-animal models [11]–[18]. Mitochondrial defects include altered morphology, transport, dynamics and bioenergetics [19]–[23]. In mutant SOD1-linked ALS, mitochondrial alterations may be partially due to the increased association of mutant SOD1 with the outer membrane and intermembrane space of the mitochondria [23]–[25]. As mitochondria are both the main producers and the targets of reactive oxygen species (ROS), increased mitochondrial ROS production may lead to mitochondrial dysfunction and cell death. Accordingly, we have shown accelerated death and mitochondrial pathology in hSOD1^G93A^ mice with decreased antioxidant defenses [26]. On the other hand, mitochondrial-targeted antioxidants can confer protection in different models of mutant hSOD1 toxicity [18], [27], [28]. Similarly, blocking the interaction of mutant SOD1 with one of its mitochondrial targets, Bcl-2, restores mitochondrial function in ALS mice [29]. These observations suggest a link between protection from mutant hSOD1-mediated motor neuron degeneration and enhanced mitochondrial function.

Single electrons escaping the respiratory chain reduce molecular oxygen to form superoxide anion in the mitochondria [30]. SOD activity converts the superoxide anion into H_2_O_2_ and oxygen. Unless H_2_O_2_ is removed by the action of glutathione peroxidase or catalase, in the presence of reduced transition metals (e.g., ferrous or cuprous ions), H_2_O_2_ can be converted into the highly reactive hydroxyl radical capable of causing several oxidative-mediated modifications in biomolecules. Increased mitochondrial oxidative stress has been linked to aging and age-related pathologies. Accordingly, increased peroxide detoxification capacity in the mitochondria extends lifespan in mice, improves age-associated reduction in mitochondrial function and is protective in a mouse model of Alzheimer's disease [31], [32], [33]. Here we investigate the effect of increased mitochondrial peroxide detoxification capacity in models of mutant hSOD1-mediated motor neuron death.

## Methods

### Animals

B6.Cg-Tg(SOD1*G93A)1Gur/J [5] and B6.Cg-Tg(SOD1*G85R)148Dwc/J [34] were obtained form The Jackson Laboratory (Bar Harbor, ME). hSOD1^H46R/H48Q^ mice originally in a mixed C3H/HeJxC57Bl/6J background were provided by Dr. David Borchelt [35] and backcrossed into C57BL/6J pure background for 6 generations. mCAT mice were previously described [36] and were kindly provided by Dr. Peter Rabinovitch (University of Washington). All the transgenic lines used in this study were maintained as hemizygous. Following genotyping animals were tagged and randomly caged. End-stage was determined by the inability of the animal to right itself within 20 seconds when placed on its side. This is a widely accepted and published endpoint for life span studies in ALS-mice and guarantees that euthanasia occurs prior to the mice being unable to forage for food or water. Mice that were unable to right themselves within 20 seconds were euthanized immediately and recorded as dead for the purpose of life span studies. Animals were euthanized by CO_2_ asphyxiation and death was confirmed by verifying respiratory arrest followed by cervical dislocation. Mice were weighed two times per week and disease onset was retrospectively determined as the time when mice reached peak body weight. All animal procedures were carried out in strict accordance with the recommendations in the Guide for the Care and Use of Laboratory Animals of the NIH. The Animal Care and Use Committee of the MUSC (Animal Welfare Assurance number is A3428-01) approved the animal protocol pertinent to the experiments reported in this publication.

### Cell cultures and treatment

Primary astrocyte cultures were prepared from cortex and spinal cord of 1-day-old mice as previously described [9]. Pups were cold-anesthetized and then euthanized by decapitation. Astrocytes were plated at a density of 2×10^4^ cells/cm^2^ and maintained in Dulbecco's modified Eagle's medium supplemented with 10% fetal bovine serum, HEPES (3.6 g/L), penicillin (100 IU/mL) and streptomycin (100 µg/mL). Astrocyte monolayers were >98% pure as determined by glial fibrillary acidic protein (GFAP, an astrocytic marker) immunoreactivity and devoid of microglial cells (as reflected by the absence of CD11b-positive cells). Motor neuron cultures were prepared from 12.5-embryonic-day mouse spinal cords as previously described [37]. For co-culture experiments, motor neurons were plated on mouse astrocyte monolayers at a density of 300 cells/cm^2^ and maintained in supplemented L15 medium [9]. Motor neurons were identified with anti-neurofilament (Sigma-Aldrich, St. Louis, MO) or anti-choline acetyltransferase antibodies (Millipore, Billerica, MA) and survival was determined by counting all cells displaying intact neurites longer than 4 cells in diameter. Counts were performed over an area of 0.90 cm^2^ in 24-well plates. Primary cortical neuronal cultures were prepared from 15-embryonic-day mouse cortices as previously described [38], with minor modifications. Cells were plated at a density of 1.5×10^5^ cells/cm^2^ and maintained in Neurobasal medium supplemented with B27 and 0.5 mM Glutamine (Invitrogen, Carlsbad, CA). Cells were harvested or treated on the seventh day after plating. Cultures were >98% pure as judge by βIII-tubulin and GFAP staining. Treatments in cortical neuronal cultures were performed in Neurobasal medium supplemented with B27 Minus AO (Invitrogen) while treatments in cortical astrocyte cultures were performed in DMEM supplemented with 2%FBS. Hydrogen peroxide (H_2_O_2_) was diluted in Dulbecco's phosphate-buffered saline and applied to the cultures at the indicated final concentrations. Survival was assayed 24 hs later by determining the release of lactate dehydrogenase (LDH) using the CytoTox Non-Radioactive Cytotoxicity Assay kit (Promega, Madison, WI). All cell culture experiments were conducted in a tri-gas incubator with 5%CO_2_ and 5%O_2_.

### Mitochondrial ROS

7DIV E15 cortical neurons were treated for 2 hs with 20 µM H_2_O_2_ or 20 µg/ml antimycin A (AA). Confluent astrocyte monolayers were treated for 3 hs with 200 µM H_2_O_2_ or 25 µg/ml AA. Following treatments, duplicate sets of cells were incubated for 30 min in Hank's balanced salt solution with 4 µM MitoSox (Invitrogen) or 0.2 µM MitoTracker Green (Invitrogen). Mitochondrial reactive oxygen species production (MitoSox, Ex/Em: 530/590 nm) was corrected by mitochondrial content (MitoTracker, Ex/Em: 485/530 nm).

### Mitochondrial isolation and catalase activity assay

Mitochondria from primary cells were isolated by differential centrifugation in a buffer containing 10 mM Tris, 1 mM EDTA, 0.32 M Sucrose and 0.2 mg/ml digitonin. Mitochondria from the spinal cord were isolated in a discontinuous Percoll gradient (method B, [39]). LDH activity was used as a cytoplasmic marker to assess the purity of the mitochondrial fraction. These mitochondrial preparations routinely yield mitochondrial fractions with less than 1.5% of the total LDH activity assayed in the crude sample. Mitochondrial fractions and total tissue were lysed in 50 mM potassium phosphate buffer pH 7.8 plus 1× complete protease inhibitor cocktail-EDTA free (Roche, Indianapolis, IN). Catalase activity was measured using the Amplex red catalase assay kit (Invitrogen). Catalase activity was corrected by protein concentration determined with BCA protein assay (Thermo Scientific, Rockford, IL), and expressed as mU per mg of protein.

### Respirometry Assay

Mitochondrial oxygen consumption rate (OCR) was determined by the addition of the complex I and complex III inhibitors rotenone (2 µM) and antimycin A (100 nM) to the cells, ATP-synthesis coupling efficiency was determined by addition of the ATP synthase inhibitor oligomycin (2 µM) and maximal respiratory capacity was determined by addition of carbonyl cyanide 4-trifluoromethoxy-phenylhydrazone (FCCP, 1 µM). The OCR measurements were performed using a Seahorse Bioscience XF-96 instrument (Seahorse Bioscience, North Billerica, MA). Astrocytes were plated in the XF-96 plates at a density of 5×103 cells per well and used a week later when confluent. The sensor cartridge was placed into the calibration buffer supplied by Seahorse Biosciences to hydrate. On the day of the experiment, the cell media was removed from the wells and well washed with DMEM media containing 5.6 mM glucose 100 nM insulin and 1% FBS one time. The DMEM media (150 µL) was then placed into the wells and warmed to 37°C in an incubator. The injection ports of the sensors were filled with 25 µL of reagent in media. The sensor was then placed into the XF-96 instrument and calibrated. After calibration, the calibration fluid plate was replaced with the cell plate. The measurement cycle consisted of a 2 min mix and a 3 min measurement. Four basal rate measurements were followed with injections and each injection is followed by four measurement cycles. The consumption rates were calculated from the continuous average slope of the O_2_ decreases using a compartmentalization model that accounts for O_2_ partitioning between plastic, atmosphere, and cellular uptake [40]. For any one genotype, the rates from an average of 15 wells were used. Average basal rates are the averages of the 3^rd^ and 4^th^ basal rate measurements and average uncoupled rates were the averages of the 1^st^ and 2^nd^ rates after FCCP injection.

### Western blot analysis

Protein samples were resolved on SDS–polyacrylamide gels and transferred to Hybond-P membranes (Amersham, Pittsburgh, PA). Membranes were blocked for 1 h in TBS, 0.1% Tween-20 and 5% BSA, followed by an overnight incubation with primary antibody diluted in the same buffer. After washing with 0.1% Tween in TBS, the membranes were incubated with peroxidase-conjugated secondary antibody (Amersham) for 1 h, and then washed and developed using the ECL chemiluminescent detection system (Amersham). A human specific rabbit anti-hSOD1 (clone EPR1726, Epitomics, CA) was used to probe for hSOD1. Densitometric analyses were performed using the NIH Image software and normalized against the signal obtained by re-probing the membranes with anti-actin (clone AC-15, Sigma-Aldrich).

### Statistical analysis

Each experiment was performed in duplicate and repeated at least three times. Groups of at least three animals were used for biochemical analysis and all data are reported as mean ± SD. Survival and onset data was analyzed with Kaplan-Meier curves and log rank test. Multiple group comparison was performed by one-way ANOVA with Bonferroni's post-test, when comparing the effect of genotype and treatments two-way ANOVA was used followed by Bonferroni's post-test and differences were declared statistically significant if *p*<0.05. All statistical computations were performed using Prism 6.0 (GraphPad Software, San Diego, CA).

## Results

### Mitochondria-targeted catalase confers resistance to oxidative stress in neurons and astrocytes

**Figure 1:**
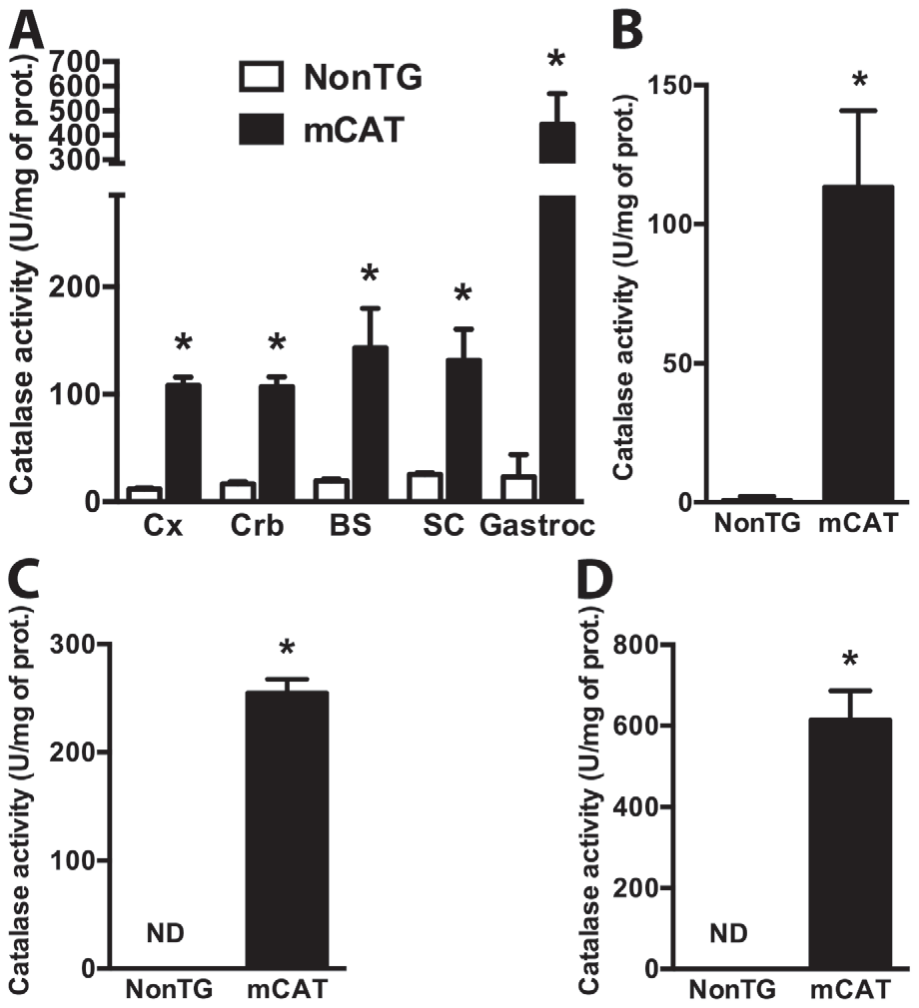
Increased mitochondrial catalase activity in the central nervous system from mCAT mice. A) Catalase activity in different regions of the central nervous system and gastrocnemius muscle (Gastroc) from 30-day-old non-transgenic (NonTG) and mCAT animals. Cx, brain cortex; Crb, cerebellum; BS, brain stem; and SC, spinal cord. B) Catalase activity in mitochondria isolated from the spinal cord of 30 days old NonTG and mCAT animals. C) Catalase activity in mitochondria isolated from primary E15 cortical neurons and D) primary cortical astrocytes obtained from NonTG and mCAT mice. For all panels, each data bar represents the mean ± SD of at least three independent experiments. *Significantly different from NonTG control (p<0.05).

**Figure 2:**
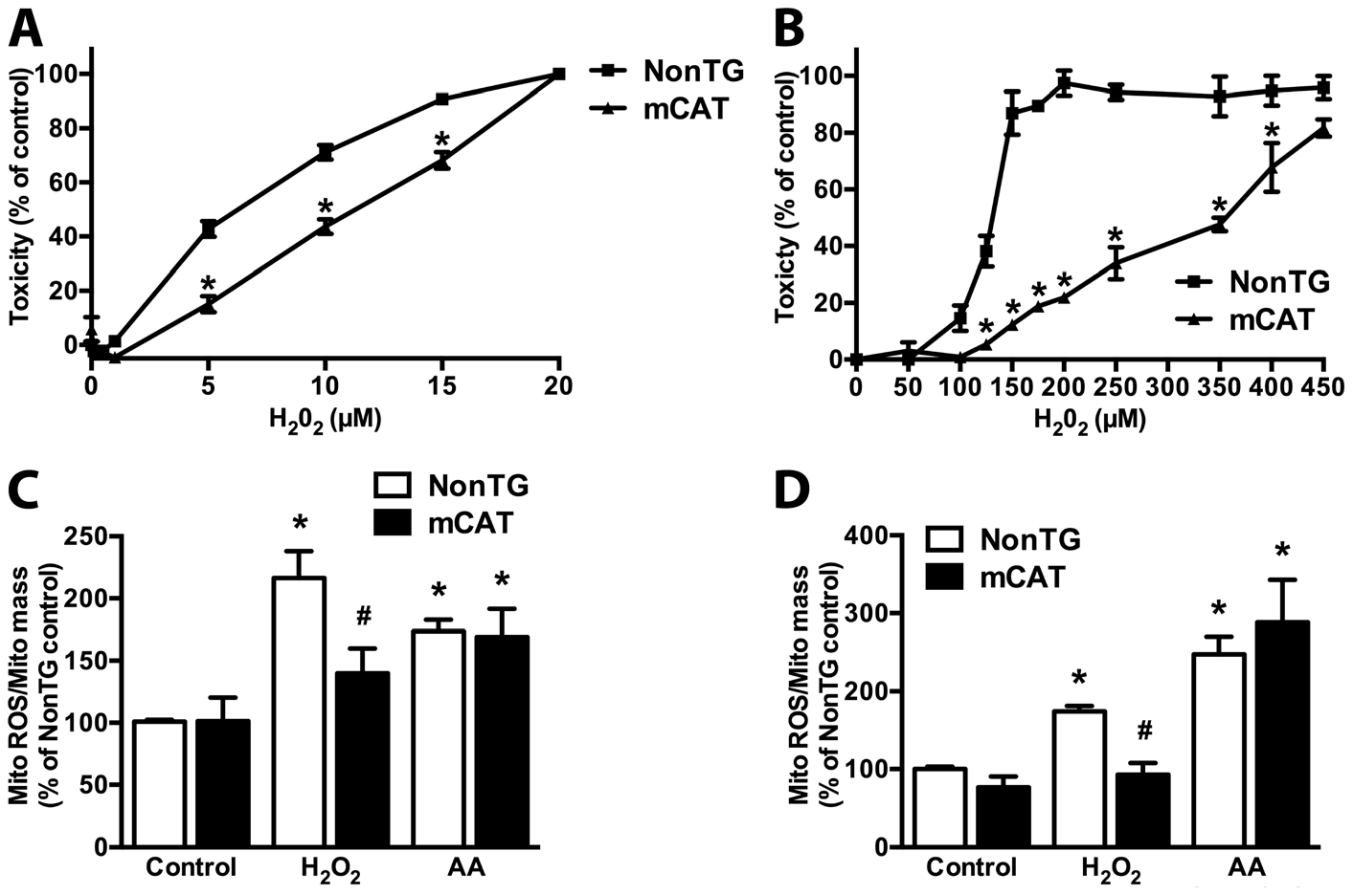
Overexpression of mitochondria-targeted catalase confers resistance against peroxide toxicity in primary cortical neurons and astrocytes. A) Cortical neuronal cultures were treated with the indicated concentrations of hydrogen peroxide (H_2_O_2_) and 24 hs later toxicity was assessed by LDH release. Data is expressed as percentage of the respective control. B) Confluent cortical astrocyte monolayers were treated with the indicated concentrations of H_2_O_2_ and 24 hs later toxicity was assessed by LDH release. Data is expressed as percentage of the respective control. C) Cortical neurons were treated with H_2_O_2_ or antimycin A (AA) and mitochondrial reactive oxygen species (MitoROS) production was determined. MitoROS was corrected by mitochondria content. Data is expressed as percentage of the non-transgenic (NonTG) control. D) Confluent astrocyte monolayers were treated with H_2_O_2_ or antimycin A (AA) followed by MitoROS and mitochondria content determination. Data is expressed as percentage of the NonTG control. For all panels, each data bar represents the mean ± SD of at least three independent experiments. *Significantly different from NonTG control (p<0.05). # Significantly different from NonTG H_2_O_2_-treated.

The transgenic mCAT mice used in this study constitutively over-express a catalase targeted to the mitochondria. Figure 1A shows total catalase activity in different regions from the central nervous system (CNS) and in gastrocnemius muscle. In order to determine if the increase in catalase activity was organelle-specific, we isolated mitochondria from the spinal cord of non-transgenic and mCAT mice and found a significant increase in catalase activity (Figure 1B). Note that although the protocol used for mitochondrial isolation typically results in mitochondrial fractions with less than 1.5% of the total LDH activity in the crude sample, traces of catalase activity were detected in the non-transgenic spinal cord mitochondria. When compare to non-transgenic controls, which display no detectable mitochondrial catalase activity, high levels of mitochondrial-specific catalase activity were detected in primary mCAT neurons and astrocytes (Figure 1C, D). mCAT primary neurons and astrocytes displayed increased resistance to oxidative stress as reflected by reduced vulnerability to H_2_O_2_ treatment (Figure 2A, B). In order to determine if decreased mitochondrial oxidative stress contributed to the protection observed in mCAT cells, the fluorescent probe MitoSox was used. MitoSox is selectively accumulated in mitochondria and oxidized as a function of ROS generation [41]. MitoTracker Green was utilized in parallel to quantify total mitochondrial mass, as this probe selectively stains mitochondria regardless of mitochondrial membrane potential [42]. Treatment of non-transgenic neuronal and astrocyte cultures with H_2_O_2_ induced a significant increase in mitochondrial ROS, which was prevented by mCAT over-expression (Figure 2C, D). Inhibition of the mitochondrial electron transfer chain by antimycin A was used as control for increased mitochondrial ROS production.

### Mitochondrial-specific catalase over-expression reverts the neurotoxicity of hSOD1^G93A^ astrocytes

**Figure 3:**
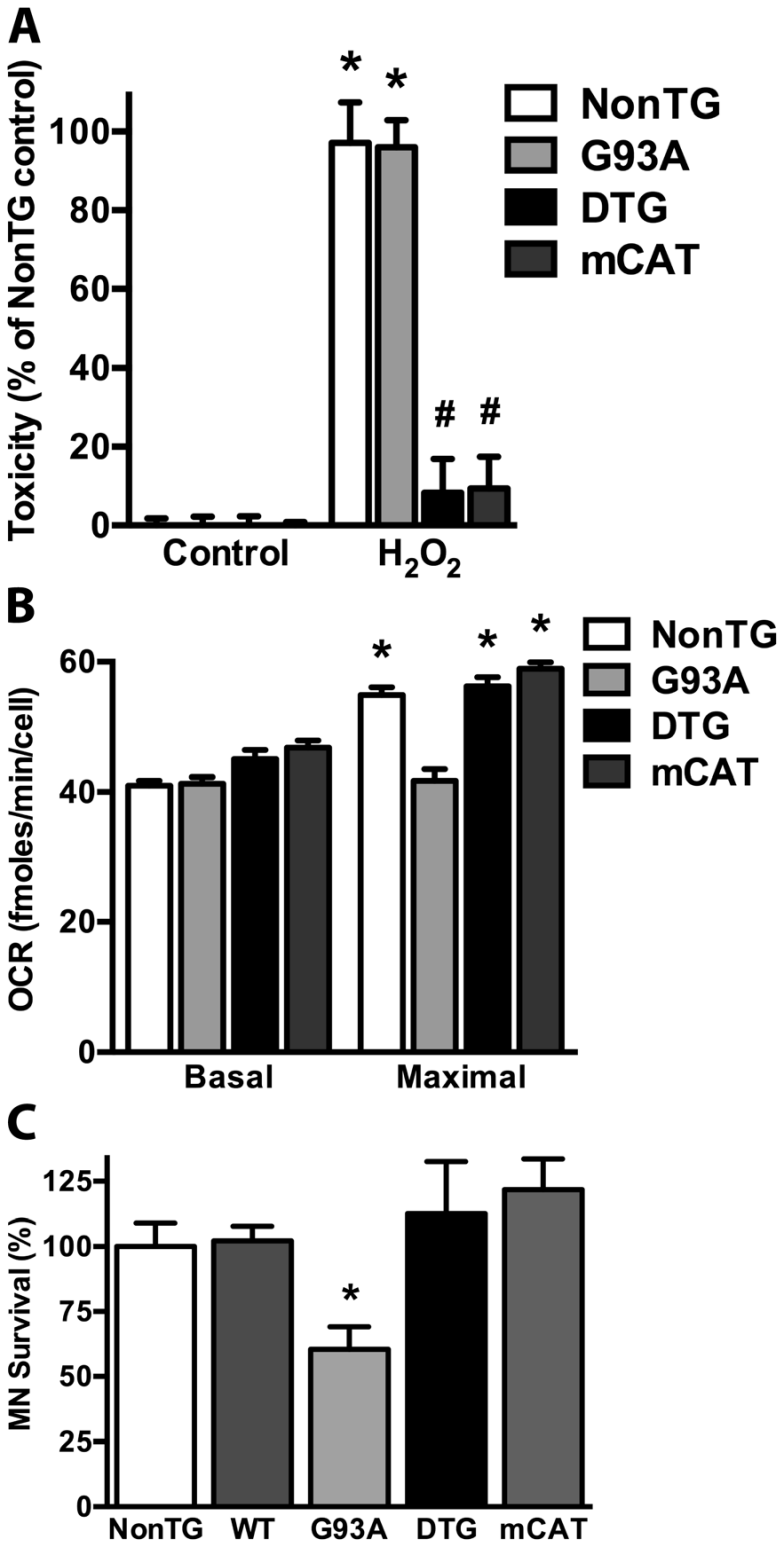
Overexpression of mitochondria-targeted catalase reverts mitochondrial dysfunction in spinal cord hSOD1^G93A^ astrocytes and reverts their toxicity towards motor neurons. A) Confluent astrocyte monolayers of the indicated genotype were treated with 200 µM hydrogen peroxide (H_2_O_2_) and 24 hs later toxicity was assessed by LDH release. Data is expressed as percentage of the respective control. NonTG, Non-transgenic astrocytes; G93A, hSOD1^G93A^ astrocytes; DTG, hSOD1^G93A^/mCAT double transgenic astrocytes. *Significantly different from NonTG control (p<0.05). # Significantly different from NonTG H_2_O_2_-treated (p<0.05). B) Oxygen consumption rate (OCR) determined for basal conditions or maximal respiration (FCCP 1 µM) in confluent spinal cord astrocyte monolayers of the indicated genotypes. *Significantly different from the respective basal OCR (p<0.05). C) Purified motor neurons from non-transgenic E12.5 mice were co-cultured over spinal cord astrocyte monolayers obtained from NonTG or transgenic mice over-expressing wild-type hSOD1 (WT), hSOD1^G93A^ (G93A), hSOD1^G93A^/mCAT (DTG) or mCAT. Motor neuron survival was assessed 72 hs later. Motor neuron loss observed in co-cultures with G93A astrocytes was prevented by catalase overexpression in DTG astrocytes. * Significantly different from NonTG control (p<0.05). For all panels, data are expressed as the mean ± SD of at least three independent experiments.

Over-expression of mitochondria-targeted catalase also conferred increased resistance against oxidative stress to astrocytes co-expressing the ALS-linked mutant hSOD1^G93A^ (double transgenic, DTG, Figure 3A). In contrast to the trophic support provided by non-transgenic astrocytes, astrocytes isolated from hSOD1^G93A^ mice induce approximately a 40% decrease in co-cultured motor neuron survival [9], [10]. Since mitochondrial dysfunction in mutant hSOD1-expressing astrocytes has been linked to this toxic phenotype [18], we employed a respirometric assay to determine the effect of hSOD1^G93A^ over-expression in oxygen consumption rates (OCR) of confluent spinal cord astrocyte cultures (Figure 3B). While, basal oxygen consumption was not significantly affected by mutant hSOD1, the reserve respiratory capacity of hSOD1^G93A^ astrocytes was significantly decreased, as reflected by decreased maximal OCR following FCCP addition (that uncouples oxidative phosphorylation from ATP synthesis). No differences were detected in ATP-synthesis coupled or non-mitochondrial OCR (not shown). Over-expression of mitochondria-targeted catalase restored the reserve respiratory capacity in hSOD1^G93A^ astrocytes and completely reversed the toxicity toward co-cultured motor neurons (Fig. 3B, C).

### Mitochondrial-specific catalase over-expression does not modify the survival of ALS-linked mutant hSOD1 mice

**Figure 4:**
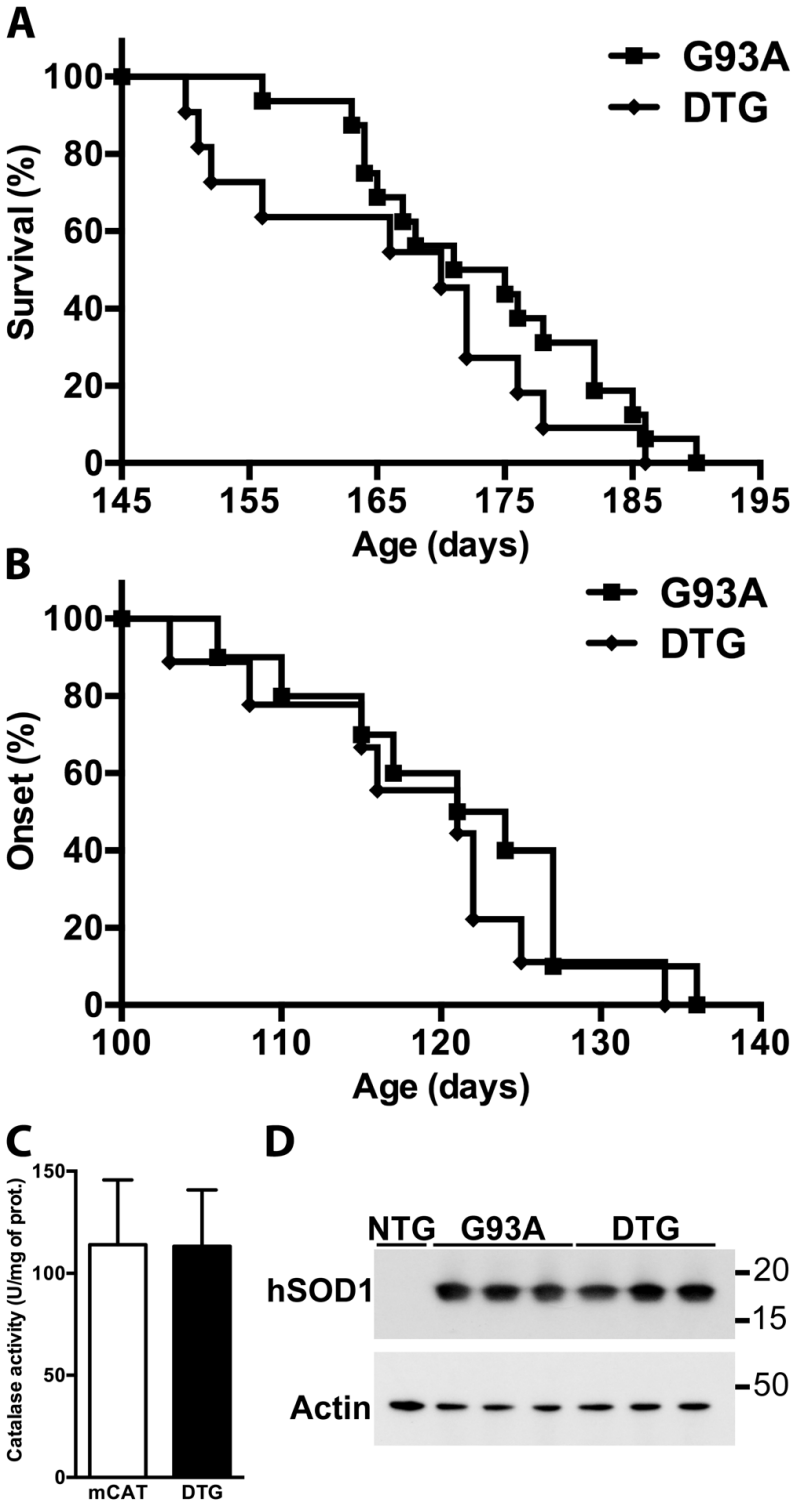
Overexpression of mitochondria-targeted catalase has no significant effect on hSOD1^G93A^/mCAT mice survival. A) Median survival in hSOD1^G93A^ (G93A) mice (173 days, n = 16) and in hSOD1^G93A^/mCAT double transgenic (DTG) mice (170 days, n = 11). Survival curves are not significantly different (χ^2^ = 1.3, p = 0.2). B) Median onset in hSOD1^G93A^ (G93A) mice (122.5 days, n = 10) and in hSOD1^G93A^/mCAT double transgenic (DTG) mice (121, n = 11). Onset curves are not significantly different (χ^2^ = 0.9, p = 0.3). C) Catalase activity in mitochondria isolated from the spinal cord of 30-day-old G93A and DTG animals. Each data bar represents the mean ± SD of at least three animals. D) hSOD1 protein expression in spinal cord extracts from 30-day-old non-transgenic (NTG), hSOD1^G93A^ (G93A) and hSOD1^G93A^/mCAT double transgenic (DTG) animals. No difference was observed in hSOD1 levels between G93A (100±8) and DTG (92±7) mice when quantified and corrected by actin levels.

Despite the protective effect conferred by mCAT expression *in vitro*, survival and disease onset were not significantly different when hSOD1^G93A^ mice were compared to hSOD1^G93A^/mCAT double-transgenic mice (Figure 4A, B). Similar results were obtained in hSOD1^G85R^ vs. hSOD1^G85R^/mCAT and hSOD1^H46R/H48Q^ vs hSOD1^H46R/H48Q^/mCAT mice (Figure S1). Transgene interactions were ruled out since the levels of mitochondrial catalase activity remained similar in mCAT and hSOD1^G93A^/mCAT littermates (Figure 4C), as well as the levels of mutant hSOD1 expression in hSOD1^G93A^ and hSOD1^G93A^/mCAT littermates (Figure 4D).

## Discussion

Although SOD1 is mainly a cytosolic protein, a fraction of wild-type SOD1 normally localizes in the mitochondria [43]. In ALS, mitochondrial alterations may be partially caused by the increased association of the mutant SOD1 with the outer membrane and intermembrane space of the mitochondria [23], [24]. Mutant hSOD1 found in the mitochondria can display aberrant catalytic chemistry that can damage key mitochondrial enzymes [44], may shift the redox state of respiratory complexes [45] or disrupt the association of cytochrome c with the inner membrane [46], causing an increase in the production of ROS. In addition, the mere increase in dismutase activity in the mitochondria may lead to higher H_2_O_2_ production unmatched by the mitochondrial antioxidant defenses. Since there is no catalase in the mitochondria from the CNS, glutathione and glutathione peroxidase are particularly important for peroxide detoxification in this organelle. In agreement, we have shown that a decrease in glutathione levels aggravates mitochondrial pathology and accelerates the course of the disease in hSOD1^G93A^ mice [26]. In this study we explored the therapeutic potential of increasing mitochondrial peroxide detoxification capacity in models of ALS by over-expressing a mitochondria-targeted catalase. While mCAT over-expression showed significant protection in several *in vitro* models, ultimately failed to extend motor neuron survival in ALS-animal models.

Neurons and astrocytes can dispose of micromolar concentrations of H_2_O_2_ with half-times in the minute range. Several enzymes are involved in H_2_O_2_ detoxification, of which catalase and glutathione peroxidases may be the most important [47], [48]. Catalase dismutates H_2_O_2_ into molecular oxygen and water, and glutathione peroxidases catalyze the reduction of H_2_O_2_ to water by the concomitant oxidation of reduced glutathione to glutathione disulfide. Increasing catalase activity in the mitochondria has the inherent advantage that its catalytic function will not depend on, or modify, the mitochondrial glutathione pool. Mitochondrial-specific catalase over-expression conferred significant resistance against H_2_O_2_ toxicity to neurons and astrocytes. In general, it is assumed that H_2_O_2_ permeates directly through membranes; although H_2_O_2_ diffusion across the plasma membrane may be facilitated by aquaporins [49]. Whether the increase in mitochondrial catalase is limiting a direct deleterious effect of exogenously added H_2_O_2_ on the mitochondria or is limiting the damage caused by a secondary increase in mitochondrial ROS following H_2_O_2_ treatment [50], remains to be established. However, it is clear that mCAT over-expression was capable to significantly decrease the production of mitochondrial ROS following H_2_O_2_ treatment (Figure 2).

Many of the different hypotheses proposed to explain motor neuron degeneration in ALS may directly or indirectly cause mitochondrial dysfunction [23], [25]. In addition, mitochondrial dysfunction and increased oxidative stress have been linked to the toxicity of mutant hSOD1 in astrocytes and neurons [18], [27]. Here we showed that primary spinal cord astrocytes isolated from hSOD1^G93A^ animals do not have the ability to increase mitochondrial respiration to meet an increase in energy demand (Figure 3B). The expression of the mutant hSOD1 could be affecting utilization of the energy substrate supply and/or impairing electron transfer. Alternatively, the lack of reserve capacity could be interpreted as if the mitochondria in these astrocytes operate at maximal capacity even under basal conditions. Decreasing peroxide accumulation in the mitochondria of hSOD1^G93A^ astrocytes restored the reserve respiratory capacity and rescued co-cultured motor neurons, providing additional evidence linking mitochondrial dysfunction and mutant hSOD1 toxicity. *In vivo*, ALS-linked hSOD1 toxicity requires damage to neuronal and non-neuronal cells in order to observe overt motor neuron degeneration [6]–[8]. Despite reversing the toxic phenotype of astrocytes *in vitro*, mCAT over-expression failed to extend survival in several mutant-hSOD1 ALS-mouse models. This observation could be explained if the mCAT over-expression fails to provide protection against the neuronal autonomous mutant hSOD1 toxicity component [51]. Decreasing glutathione levels significantly accelerates motor neuron death in hSOD1^G93A^ ALS-mice, at least in part, by aggravating mitochondrial pathology [26]. Glutathione is crucial to detoxify peroxides in the mitochondria; however, the data presented here suggest that mitochondrial peroxide detoxification may not be a critical component affected. Alternatively, changes in glutathione may be affecting the association of mutant hSOD1 with the mitochondria [26] without having a major effect on mitochondrial antioxidant defenses.

We showed here that selective mitochondria-targeted catalase over-expression improves mitochondrial antioxidant defenses and mitochondrial function in hSOD1^G93A^ astrocyte cultures, however ALS-animals did not develop the disease later nor survive longer. Since the toxicity of hSOD1^G93A^ is not limited to the mitochondria, modulation of antioxidant defenses in other subcellular compartments may also be required. Hence, while increased oxidative stress and mitochondrial dysfunction have been extensively documented in ALS, these results suggest that preventing peroxide-mediated mitochondrial damage alone is not sufficient to delay the disease.

## Supporting Information

Figure S1 **Overexpression of mitochondria-targeted catalase has no significant effect on the survival of hSOD1^G85R^/mCAT and hSOD1^H46R/H48Q^/mCAT mice.** A) Median survival in hSOD1^G85R^ mice (366 days, n = 8) and in hSOD1^G85R^/mCAT double transgenic mice (359 days, n = 7). Survival curves are not significantly different (χ^2^ = 0.23, p = 0.6). B) Median survival in hSOD1^H46R/H48Q^ mice (227 days, n = 7) and in hSOD1^H46R/H48Q^/mCAT double transgenic mice (235 days, n = 11). Survival curves are not significantly different (χ^2^ = 0.55, p = 0.4).
(TIF)
