# Supplementary figures and images for: Mitochondria-Targeted Catalase Reverts the Neurotoxicity of hSOD1G93A Astrocytes without Extending the Survival of ALS-Linked Mutant hSOD1 Mice
Source: PLoS One. 2014 Jul 23;9(7):e103438. doi: 10.1371/journal.pone.0103438 (PMC4108402; doi:10.1371/journal.pone.0103438)

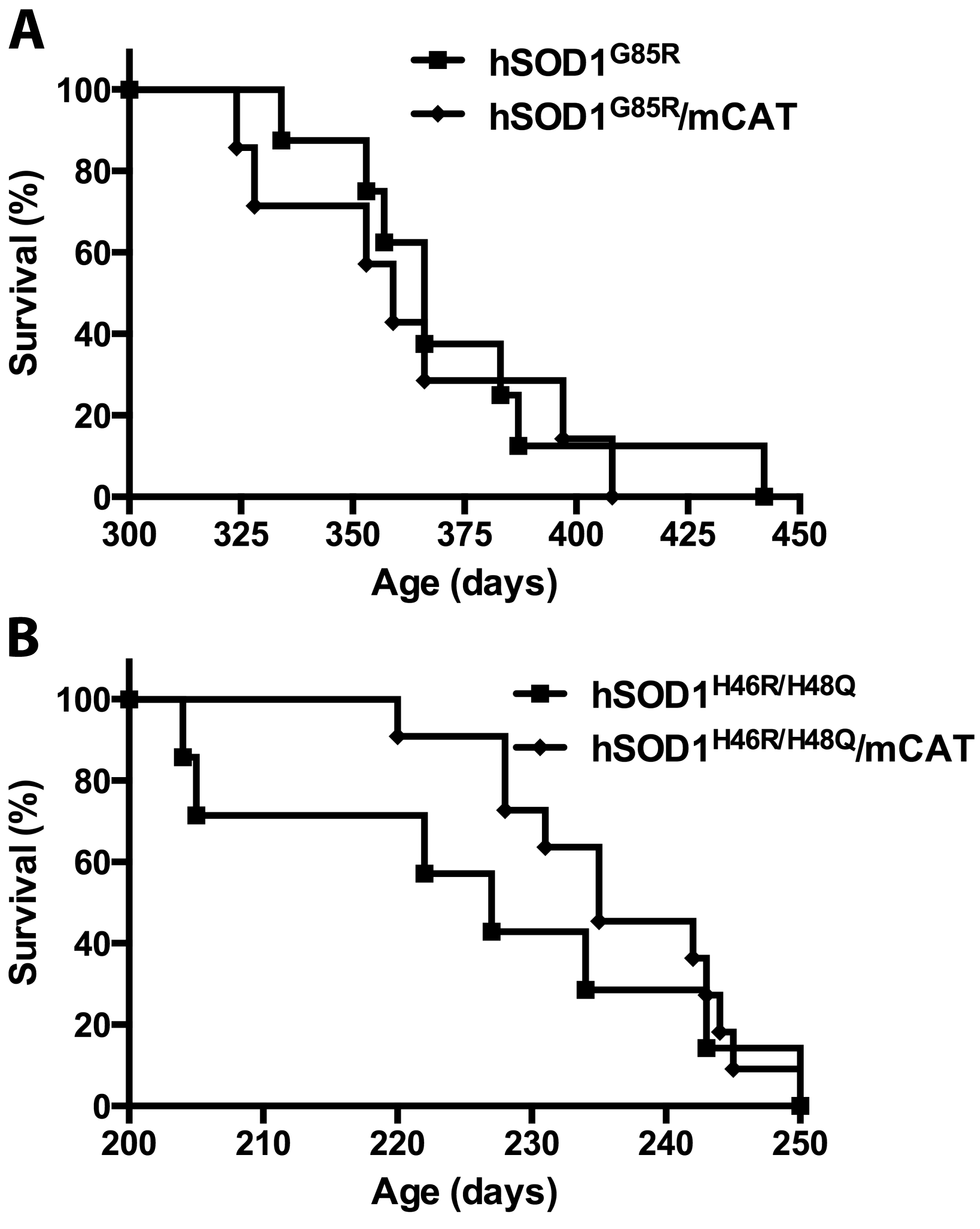

Supplement: Figure S1 — Overexpression of mitochondria-targeted catalase has no significant effect on the survival of hSOD1G85R/mCAT and hSOD1H46R/H48Q/mCAT mice. A) Median survival in hSOD1G85R mice (366 days, n = 8) and in hSOD1G85R/mCAT double transgenic mice (359 days, n = 7). Survival curves are not significantly different (χ2 = 0.23, p = 0.6). B) Median survival in hSOD1H46R/H48Q mice (227 days, n = 7) and in hSOD1H46R/H48Q/mCAT double transgenic mice (235 days, n = 11). Survival curves are not significantly different (χ2 = 0.55, p = 0.4). (TIF) [file pone.0103438.s001.tif]
